# Changes in Retinal Blood Flow in Response to an Experimental Increase in IOP in Healthy Participants as Assessed With Doppler Optical Coherence Tomography

**DOI:** 10.1167/iovs.61.2.33

**Published:** 2020-02-21

**Authors:** Stefan Puchner, Doreen Schmidl, Laurin Ginner, Marco Augustin, Rainer Leitgeb, Stephan Szegedi, Kristina Stjepanek, Nikolaus Hommer, Martin Kallab, René Marcel Werkmeister, Leopold Schmetterer, Gerhard Garhofer

**Affiliations:** 1 Center for Medical Physics and Biomedical Engineering, Medical University of Vienna, Vienna, Austria; 2 Department of Clinical Pharmacology, Medical University of Vienna, Vienna, Austria; 3 Department of Ophthalmology, Vienna Institute for Research in Ocular Surgery–Karl Landsteiner Institute, Hanusch Hospital, Vienna, Austria; 4 Singapore Eye Research Institute, Singapore National Eye Centre, Singapore; 5 Lee Kong Chian School of Medicine, Nanyang Technological University, Singapore; 6 Ophthalmology and Visual Sciences Academic Clinical Program, Duke-NUS Medical School, Singapore; 7 SERI-NTU Advanced Ocular Engineering (STANCE), Singapore, Singapore; 8 Institute of Ophthalmology, Basel, Switzerland

**Keywords:** ocular perfusion pressure, suction cup, retinal blood flow, Doppler optical coherence tomography, intraocular pressure, autoregulation

## Abstract

**Purpose:**

Blood flow autoregulation is an intrinsic mechanism of the healthy retinal vasculature to keep blood flow constant when ocular perfusion pressure (OPP) is changed. In the present study, we set out to investigate retinal blood flow in response to an experimental decrease in OPP in healthy participants using Doppler optical coherence tomography.

**Methods:**

Fifteen healthy participants aged between 22 and 31 years (mean, 27 ± 3 years) were included in the present open study. IOP was increased stepwise via the suction cup method to induce a decrease in OPP. Retinal blood flow in arteries and veins was assessed using a custom-built Doppler optical coherence tomography system and pressure–flow relationships were calculated to assess autoregulation.

**Results:**

Suction cup application induced a pronounced increase in IOP with a maximum value of 50.5 ± 8.0 mm Hg at the highest level of suction. Pressure–flow relationships revealed that blood flow was autoregulated until the OPP was decreased by approximately 21 mm Hg and started to decrease significantly when the OPP was reduced by 30 mm Hg. Retinal blood flow at the last suction period decreased at a maximum of –57.0 ± 22.3% and 65.2 ± 15.4% in retinal arteries and retinal veins, respectively. These changes in retinal blood flow were less pronounced than the decrease in OPP (–75.2 ± 19.2%), indicating retinal autoregulation.

**Conclusions:**

The results of the present study confirm that retinal blood flow is autoregulated in response to changes in the OPP. Doppler optical coherence tomography has the potential to become a clinical tool for the investigation of retinal blood flow autoregulation in the future, because of its ability to assess the blood velocities and diameter of the retinal vessels parallel and therefore also their blood flow in absolute values. (Clinicaltrials.gov number NCT03398616)

Autoregulation is a key feature of the healthy ocular vasculature to ensure sufficient delivery of oxygen and nutrition to the eye during fluctuations of blood pressure or IOP.^1^ As such, it has been shown that in healthy humans as well as experimental animals, a decrease in ocular perfusion pressure (OPP) does not translate to a concomitant reduction in choroidal and optic nerve head blood flow.^2^^–^^10^ There is general agreement that this is caused by the regulatory capacity of the vascular beds, which counteract the change in perfusion pressure^11^ and ensure a stable blood flow despite changes in IOP or systemic blood pressure. Although autoregulatory properties have been consistently shown for the optic nerve head and to a certain extent also for the choroidal circulation, the investigation of retinal autoregulatory properties is hampered by technical limitations and the absence of a gold standard to assess retinal blood flow in humans.^12^ In this context, the assessment of volumetric blood flow in retinal vessels is challenging, especially during experimentally introduced changes in OPP. Although blood flow in retinal arteries and veins has been investigated for many years using techniques such as laser Doppler velocimetry or laser speckle flowgraphy,^13^^,^^14^ it recently also became possible to study the retinal circulation using Doppler optical coherence tomography (D-OCT). This technique has the advantage of providing retinal blood velocity as well as vessel diameter in absolute values within one measurement,^15^ whereas most previous studies investigating retinal autoregulatory properties in humans measured retinal blood velocity and diameters separately or used vessel diameters only.^16^^–^^18^

In the present study, we take advantage of this functional imaging technique and set out to investigate retinal blood flow regulation in response to an experimental decrease in OPP in healthy participants. For this purpose, OPP was decreased by stepwise elevation of IOP using the suction cup method. The response of the retinal circulation to a decrease in OPP was measured using D-OCT and pressure flow relationships were calculated to assess retinal autoregulation.

## Methods

### Participants

The present study was performed in adherence to the Declaration of Helsinki and the Good Clinical Practice guidelines of the European Union. The study protocol was approved by the Ethics Committee of the Medical University of Vienna. All study participants gave informed consent before participation in the trial.

During the 4 weeks before the first study day, a screening examination was carried out that included recording of medical history, a pregnancy test in females of childbearing potential, and a full ophthalmologic examination (best-corrected visual acuity, slit lamp examination, measurement of IOP and indirect fundoscopy). Only participants with ametropia of less than 1 diopter and no evidence of any eye diseases were included. In the 3 weeks before the first study day and during the course of the study, no blood donation and no intake of concomitant medication was allowed, except contraceptives. During the week after completion of the study, a follow-up safety ophthalmologic examination was performed.

### 

### Noninvasive Measurement of Systemic Hemodynamics

Systolic and diastolic blood pressures, as well as the mean arterial pressure (MAP) were measured on the upper arm by an automated oscillometric device. Pulse rate was automatically recorded from a finger pulse oximetry device (Infinity Delta, Draeger, Luebeck, Germany).

### IOP and OPP

IOP was measured using a slit lamp mounted Goldmann applanation tonometer. Before each measurement, one drop of oxybuprocaine hydrochloride combined with sodium fluorescein was used for local anesthesia of the cornea. OPP was estimated as 2/3 MAP–IOP.^19^

### Suction Cup Method

For IOP elevation, a suction cup device was used.^20^ After topical anesthesia with oxybuprocaine hydrochloride eye drops, a rigid plastic suction cup with a diameter of 11 mm was placed on the temporal sclera with the anterior edge at least 1 mm away from the limbus. The suction cup was connected to an automatic suction pump through plastic tubing, which was used to stepwise increase the vacuum from 25 to 125 mm Hg, resulting in an IOP increase. No additional pressure was required for the suction cup to stay on the ocular tissue because the created vacuum was sufficient.

### Doppler Optical Coherence Tomography

For the measurement of retinal blood flow, a custom-built dual-beam Fourier domain D-OCT system coupled to a fundus camera that has been described in detail previously was used.^15^^,^^21^ Measurements were performed in one artery and one vein with an approximate distance of one disc diameter from the rim of the optic nerve head. Briefly, the vessels are illuminated with two orthogonally polarized laser beams that stem from the same superluminescent diode under a defined angle Δα in between the vessels to overcome the problem of angle ambiguity. For detection of the spectral information of the two orthogonally polarized laser beams, the system consists of two spectrometers providing two OCT images yielded from the two different angles of incidence of the probe beam. The phase changes in these channels are different according to the different Doppler angles. The central wavelength of the laser is at λ_0_ = 838.8 nm with a spectral bandwidth of 54.0 nm, resulting in an axial resolution of approximately 6 µm and a lateral resolution of approximately 20 µm. A bidirectional OCT system is used because, in D-OCT, knowledge on the angle between the incident light beam and the vessel is required to calculate the absolute blood velocity.^22^ When two light beams are used, however, knowledge of the Doppler angle is not necessary as long as Δα is small.^15^

Vessel diameters were extracted from the phase images as described previously.^23^ Briefly, in the phase image, a phase contrast between moving red blood cells and static tissue is observed. To obtain vessel calibers, the outer border of these phase-shifted areas was determined manually from seven images per vessel and the mean value was used, taking the depth range of the OCT system and the refractive index of blood into account. For extraction of retinal vessel diameter, the channel with the higher phase shift was used.

### Description of the Study Day

At the beginning of the study day, a pregnancy test was performed in females of childbearing potential. The participants were studied after topical instillation of one drop of tropicamide into the study eye.

A resting period of at least 20 minutes was scheduled before measurements were started to ensure constant hemodynamic conditions. Retinal blood flow in one vein and artery was measured at baseline using D-OCT. Thereafter, the suction cup was placed on the sclera and IOP was increased by a suction cup at levels of 25, 50, 75, 100, and 125 mm Hg for 1 minute each and retinal blood flow was measured at each step 30 seconds after reaching the target pressure. Systemic hemodynamic parameters were concomitantly measured every minute. Because it is not possible to measure IOP by applanation tonometry and retinal blood flow simultaneously, a second period of suction was scheduled 30 minutes later. During this period, the IOP was measured at each level of suction using applanation tonometry.

### Data Analysis

The effect of suction cup application at each step on the outcome parameters MAP, IOP, OPP, retinal vessel diameters, retinal blood velocity and retinal blood flow was analyzed using one-way repeated measures ANOVA and a *P* value of less than 0.05 in comparison with baseline values was considered as the level of significance.

In addition, data were expressed as OPP and flow values to show pressure–flow relationships. OPP values were sorted in descending order and grouped into six categories. From a total of 180 measurements, 10 values for retinal arteries and 8 for retinal veins had to be excluded from the analysis due to insufficient target fixation. As such, on average 14 values were classified in each of the groups for the pressure–flow relationships in retinal arteries and veins. A statistically significant deviation from baseline flow was defined when the 95% confidence interval did not overlap with the baseline value anymore.^24^^,^^25^ Statistical analysis was carried out using CSS Statistica for Windows (Statsoft Inc., Version 6.0, Tulsa, OK).

## Results

Fifteen healthy participants aged between 22 and 31 years (mean, 27 ± 3 years) were included and finished the study according to the protocol. Six of them were male and nine were female.

### IOP and OPP 

**Figure 1.**
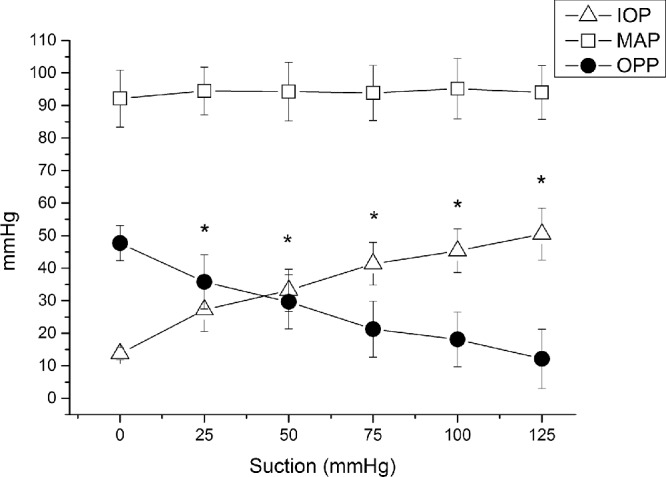
Changes in IOP, MAP and OPP during application of the suction cup. Data are presented as mean ± SD (*n* = 15). *Significant changes versus baseline for IOP and OPP.

**Figure 2.**
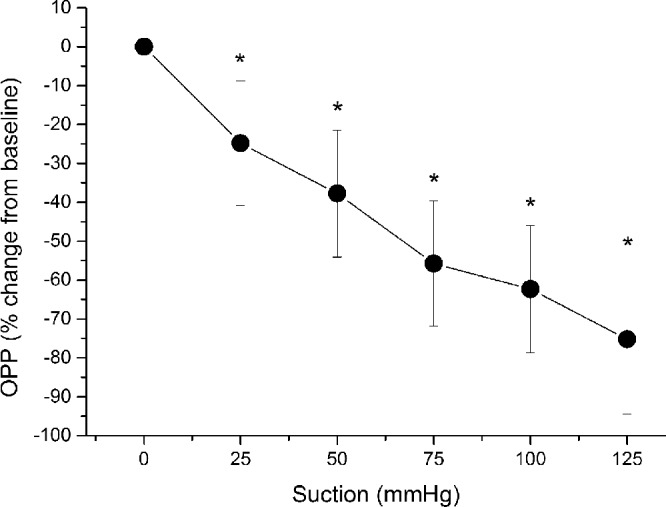
Relative change in OPP during application of the suction cup. Data are presented as mean ± SD (*n* = 15). *Significant changes versus baseline.

IOP at baseline was 14 ± 2 mm Hg. Application of the suction cup was well-tolerated except for the occurrence of transient mild conjunctival hyperemia. As expected, suction cup application induced a pronounced increase in IOP at all five pressure steps (*P* < 0.001 for each step), with a maximum value of 50.5 ± 8.0 mm Hg at the highest level of suction. The MAP did not change during the artificial increase of IOP (*P* > 0.40 at each step). As shown in Figure 1, OPP decreased with each pressure step starting from an OPP of 47.7 ± 5.4 mm Hg at baseline finally reaching a level 12.1 ± 9.1 mm Hg at the highest level of suction (*P* < 0.001 for each step). The stepwise relative decrease in OPP is depicted in Figure 2 showing a maximum decrease of –75.2 ± 19.2% from baseline (*P* < 0.001) at the highest suction level.

### Retinal Blood Flow

**Figure 3.**
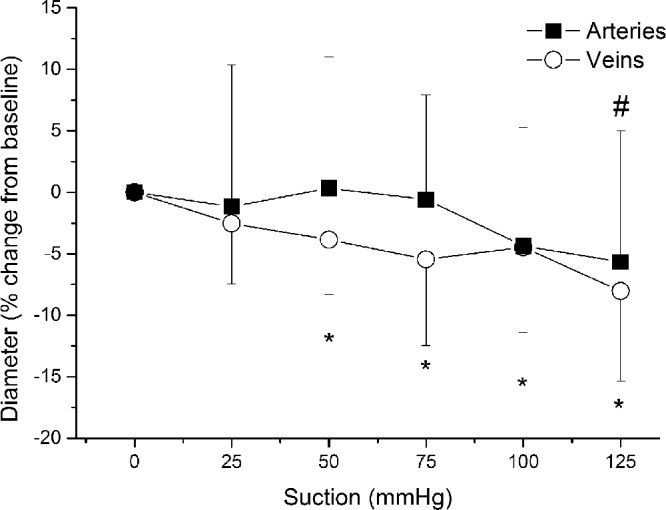
Relative change in arterial and venous vessel diameter during application of the suction cup. Data are presented as mean ± SD (*n* = 15). *Significant changes versus baseline for retinal veins; #significant changes versus baseline for retinal arteries.

**Figure 4.**
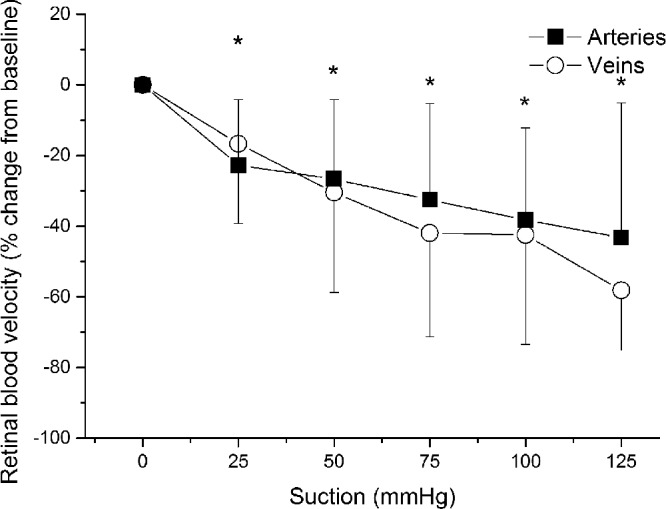
Relative change in arterial and venous blood velocity during application of the suction cup. Data are presented as mean ± SD (*n* = 15). *Significant changes versus baseline for retinal arteries and veins.

Retinal arterial vessel calibers did not change compared with baseline until the highest level of suction was reached. As shown in Figure 3, at this final suction level, retinal arterial diameters were decreased by –5.7 ± 10.6% (*P* < 0.05 vs. baseline). Retinal arterial blood velocity was stepwise decreased reaching its maximum reduction of 43.3 ± 38.1% at the highest level of suction (*P* < 0.001 vs. baseline; Fig. 4). Retinal arterial blood flow decreased significantly and was –57.0 ± 22.3% lower than at baseline at the last suction period (*P* < 0.001).

The venous diameter started to significantly decrease at the second suction step and continued to decrease with a maximum decrease of –8.0 ± 7.3% (*P* < 0.001) at the highest level of suction (Fig. 3). As shown in Figure 4, the decrease in retinal venous blood velocity was more pronounced than the decrease in vessel calibers with a maximum effect at the end of the experiments (–58.2 ± 17.0%; *P* < 0.001 vs. baseline). As for retinal arteries, venous blood flow was significantly reduced after application of the suction cup. The relative decrease in retinal blood flow was, however, less pronounced than that in OPP at all levels of suction (maximum decrease in retinal venous blood flow, –65.2 ± 15.4%; *P* < 0.001 each).

### Pressure–Flow Relationship

**Figure 5.**
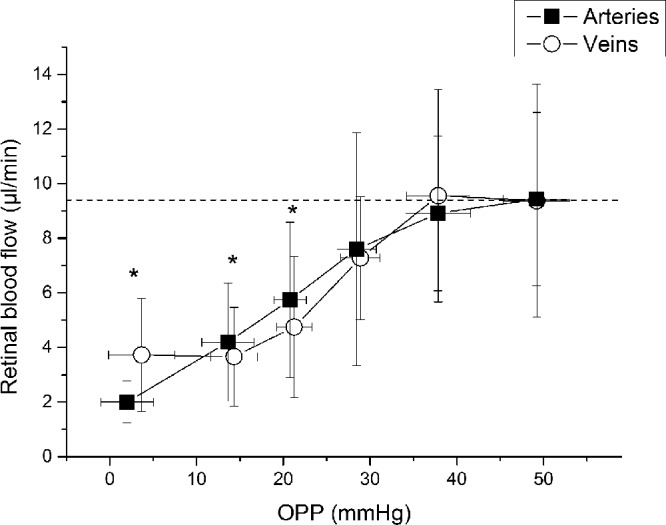
Pressure–flow relationship for retinal arteries and veins determined by categorized OPP and retinal blood flow during application of the suction cup. Data were sorted into groups of 15 values, each according to ascending OPP. Data are presented as mean ± SD (*n* = 15). *Significant changes versus baseline for retinal arteries and veins.

To assess the autoregulatory range, pressure–flow relationships were calculated by grouping flow data according to descending OPP values. As shown in Figure 5, no significant change in blood flow in arteries or veins was observed until the OPP was decreased by approximately 20 mm Hg (from 49 to 28 mm Hg), indicating blood flow autoregulation. Blood flow in arteries and veins started to decrease significantly when OPP was reduced by approximately 30 mm Hg (from 49 to 21 mm Hg). Over the entire range of the pressure–flow relationship, the decrease in blood flow was also less pronounced than the decrease in OPP.

**Figure 6.**
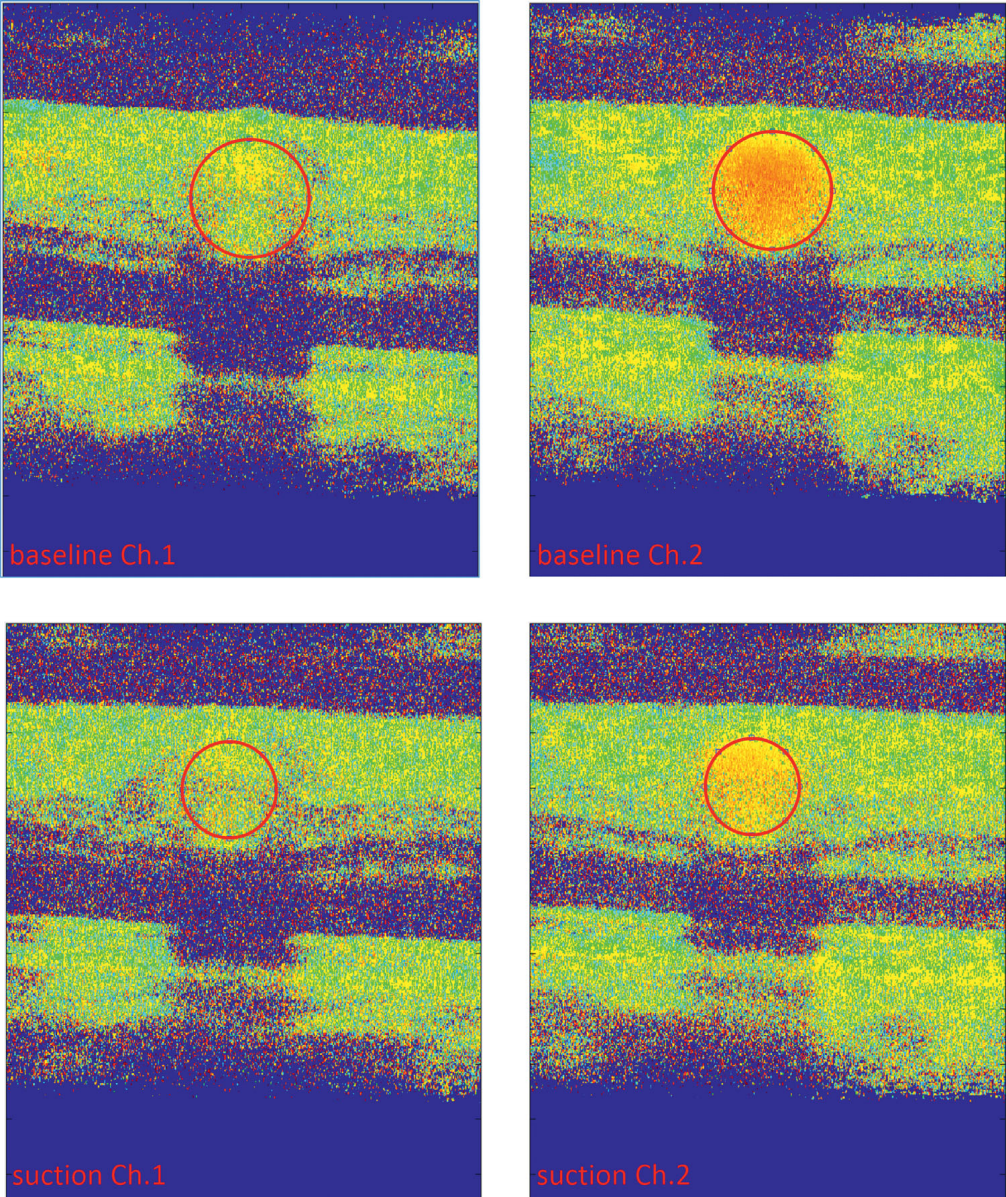
Representative summed phase images of both channels as acquired with the bidirectional Doppler-OCT at baseline (*top*) and during the highest suction level (*bottom*). As for channel 1, the angle of the measurement beam was almost perpendicular to the vessel and the observed phase shift in channel 1 is only small. Vessel diameter was determined based on the channel with the higher phase shift (channel 2). The vessels of interest are marked in *red* and decrease in size upon suction.

A sample image of the Doppler-OCT measurements at baseline and the highest level of suction in one participant is given in Figure 6.

## Discussion

This is the first study investigating retinal blood flow autoregulation using D-OCT to measure volumetric retinal blood flow. Our results confirm the autoregulatory properties of the retinal circulation, showing that retinal blood flow remains stable until OPP values of 20 mm Hg below baseline and starts to significantly decrease when perfusion pressure is decreased by approximately 30 mm Hg. The response of both retinal blood velocity and blood flow to an increase in IOP was less pronounced than the decrease in OPP, indicating a retinal autoregulatory response.

Data regarding retinal blood flow autoregulation are sparse and their collection hampered by the technical difficulties to assess retinal blood flow. In most of the available studies, retinal blood flow was either determined by separate assessments of blood velocity and vessel calibers or by using either diameter or velocity data only to estimate perfusion.^16^^–^^18^^,^^26^ As such, it has been shown in a previous experiment using laser Doppler velocimetry to assess blood velocity combined with measurements of vessel diameter data that retinal blood flow is autoregulated until IOP increases above 30 mm Hg.^17^

Other experimental evidence using fluorescein angiography to estimate retinal perfusion also reported failure of retinal blood flow autoregulation at IOP levels of greater 30 mm Hg.^18^ These values are lower than those found for the optic nerve head, where blood flow also seems to be relatively stable until IOP values of 40 mm Hg or higher are reached.^7^^,^^8^^,^^11^ The reason for this difference is not entirely clear, but could be related to the fact that optic nerve head blood flow autoregulation in previous studies was assessed in the capillary bed using laser Doppler flowmetry, whereas in the present study retinal blood flow was measured in major retinal arteries and veins.

In the current study, pressure–flow relationships have been used to assess the autoregulatory capacity of the retinal circulation. This approach has been used previously to determine vascular autoregulatory response in the choroid^2^^,^^4^^,^^5^^,^^27^ and the optic nerve head.^9^^,^^28^^–^^30^ For this analysis, blood flow values are grouped according to the OPP values. This grouping is necessary because, when increasing the IOP by a stepwise increasing suction force, the resulting OPP is not only dependent on the suction force applied, but also on MAP, baseline IOP and the biomechanical properties of the tissue. The stiffness of the ocular tissues determines how much the IOP increases at a certain level of suction. Thus, the same suction force does not necessarily translate into the same change in OPP. Consequently, is not possible to determine the autoregulatory range by simply comparing the blood flow response at different suction forces. Sorting blood flow by OPP overcomes this problem and allows for the comparison of blood flow changes at similar OPP changes. Hence, this way of analyzing data is more suitable to determine the autoregulatory range of a vascular bed. Based on this analysis, our results show a lower limit for autoregulation of the retinal circulation at an OPP decrease of 30 mm Hg, which is in the same pressure range as previously published results.^17^

Further, the finding that retinal blood flow shows an autoregulatory response is also underlined by the fact that over the entire range of different OPPs, the decrease in blood flow was also less pronounced than the decrease in OPP. In particular, the maximum relative decrease in OPP (–75.2%; Fig. 2) was more pronounced than the relative decrease in retinal blood flow (–57.0 ± 22.3%), which again supports the concept of an autoregulatory response of the retinal vasculature.

Interestingly, the response of retinal arterial and venous calibers to an experimental increase in the IOP was different. Although the arterial diameter did not significantly change until the highest level of suction, the venous diameter started to decrease immediately after reducing the OPP (Fig. 3). Previous experiments investigating the vessel caliber in response to increased IOP report a small but significant dilation of arteries accompanied by a decreased venous diameter.^16^^,^^31^ Although arterial dilation at a lower IOP has been interpreted as a part of the autoregulatory response to keep blood flow constant despite the decrease in OPP,^16^^,^^31^ lower transmural pressure differences at high IOPs may lead to a compression of the vessel resulting in a decrease in the net vessel caliber.^16^^,^^31^ In contrast with arteries, the reason for the decreased venous diameter is less clear. It may be speculated that the decreased caliber observed in veins is caused be a passive compression effect owing to the weaker wall construction of retinal veins compared with arteries.

Our results are well-comparable with previous experiments. In the present study, we show a numerical, but not statistically significant, trend toward increased diameter in retinal arterial diameter at lower IOPs, whereas at higher IOPs a decreased vessel caliber was seen. Interestingly, in retinal veins, our data indicate a more pronounced caliber reduction compared with previously published reports. This effect could, however, be related to the different method used for diameter measurement in the current study. Whereas previous studies have used fundus images to extract diameter data, in the current study, vessel diameters were determined based on the phase image of the D-OCT. The phase image reflects the contrast between moving particles (red blood cells) and static tissue.^23^ This approach has the advantage that diameter and velocity can be measured concomitantly at the same location. However, when the velocity of moving particles is decreased, as in the present experiment, the phase noise at the vessel border of the vessel increases, which in turn may lead to a slight underestimation of vessel calibers that needs to be considered as a limitation when interpreting our results. In contrast with the technique used in the current experiment, standard methods for the assessment of vessel calibers, such as the Dynamic Vessel Analyzer, are based on measurement of the erythrocyte column within the vessels.^32^ Thus, we cannot exclude that the method used in our study led to a slight underestimation of the effect on vessel calibers in the early pressure steps and may explain the slightly more pronounced diameter changes in a previous report.^16^

The strength of our study is the custom-built D-OCT system, which was used to assess volumetric blood flow. This system has the advantage that, in comparison with other techniques, it provides absolute values for retinal blood flow in veins and arteries with one measurement, because diameters and blood velocities are assessed in parallel.

The setup used in the present study is experimental, but recently a D-OCT prototype that may soon become commercially available has been introduced.^33^ It is, therefore, reasonable to assume that retinal blood flow measurements may in the future be applicable in clinical practice. The present study indicates that D-OCT has the ability to study the physiologic behavior of the retinal vasculature in response to changes in perfusion pressure, as well as in diseases that are associated with retinal blood flow abnormalities such as glaucoma^34^ or diabetes.^35^

The present study also has some limitations. Retinal blood flow was only measured in one major retinal vein and one artery. It would have been of interest to assess total retinal blood flow, but because measurement of total retinal blood flow is still time consuming, this approach is not feasible during application of the suction cup. Further, we only measured blood flow and diameter changes in major retinal vessels. Hence, we cannot provide data regarding the blood flow and diameter changes in smaller retinal arterioles that are considered to play the most important role in the vascular resistance changes during changes in the OPP or in the microcirculation. Further studies using multimodal approaches in several vessels are required to obtain a more complete understanding of retinal autoregulation including the microcirculation. Additionally, measurements were performed 30 seconds after reaching the target pressure. We cannot fully exclude that the autoregulatory response of retinal vessels is not fully established at this time and may be more pronounced after a longer period of suction for each step.^16^ This step was, however, not possible in the current experimental setup owing to ethical considerations, which limit the maximum time of IOP increase in healthy participants.

In conclusion, the results of the present study indicate that retinal blood flow is autoregulated in response to a decrease in OPP. These findings are in good agreement with previous reports using different techniques for assessment of retinal blood flow. Given that D-OCT is easily applicable and commercial prototypes for this technology already exist, the investigation of retinal blood flow autoregulation in patients with disease may become feasible in the near future.

## References

[1] Flammer J, Mozaffarieh M Autoregulation, a balancing act between supply and demand. *Can J Ophthalmol*. 2008; 43: 317–321. 18493273 10.3129/i08-056

[2] Simader C, Lung S, Weigert G, et al. Role of NO in the control of choroidal blood flow during a decrease in ocular perfusion pressure. *Invest Ophthalmol Vis Sci*. 2009; 50: 372–377. 19124845 10.1167/iovs.07-1614

[3] Polska E, Simader C, Weigert G, et al. Regulation of choroidal blood flow during combined changes in intraocular pressure and arterial blood pressure. *Invest Ophthalmol Vis Sci*. 2007; 48: 3768–3774. 17652750 10.1167/iovs.07-0307

[4] Schmidl D, Weigert G, Dorner GT, et al. Role of adenosine in the control of choroidal blood flow during changes in ocular perfusion pressure. *Invest Ophthalmol Vis Sci*. 2011; 52: 6035–6039. 21697134 10.1167/iovs.11-7491

[5] Riva CE, Titze P, Hero M, Petrig BL Effect of acute decreases of perfusion pressure on choroidal blood flow in humans. *Invest Ophthalmol Vis Sci*. 1997; 38: 1752–1760. 9286263

[6] Weigert G, Findl O, Luksch A, et al. Effects of moderate changes in intraocular pressure on ocular hemodynamics in patients with primary open-angle glaucoma and healthy controls. *Ophthalmology*. 2005; 112: 1337–1342. 16024084 10.1016/j.ophtha.2005.03.016

[7] Riva CE, Hero M, Titze P, Petrig B Autoregulation of human optic nerve head blood flow in response to acute changes in ocular perfusion pressure. *Graefes Arch Clin Exp Ophthalmol*. 1997; 235: 618–626. 9349945 10.1007/BF00946937

[8] Pillunat LE, Anderson DR, Knighton RW, Joos KM, Feuer WJ Autoregulation of human optic nerve head circulation in response to increased intraocular pressure. *Exp Eye Res*. 1997; 64: 737–744. 9245904 10.1006/exer.1996.0263

[9] Schmidl D, Boltz A, Kaya S, et al. Comparison of choroidal and optic nerve head blood flow regulation during changes in ocular perfusion pressure. *Invest Ophthalmol Vis Sci*. 2012; 53: 4337–4346. 22661477 10.1167/iovs.11-9055

[10] Kiel JW, Shepherd AP Autoregulation of choroidal blood flow in the rabbit. *Invest Ophthalmol Vis Sci*. 1992; 33: 2399–2410. 1634337

[11] Schmidl D, Garhofer G, Schmetterer L The complex interaction between ocular perfusion pressure and ocular blood flow - relevance for glaucoma. *Experimental eye research*. 2011; 93: 141–155. 20868686 10.1016/j.exer.2010.09.002

[12] Pournaras CJ, Riva CE Retinal blood flow evaluation. *Ophthalmologica*. 2013; 229: 61–74. 23257770 10.1159/000338186

[13] Schmetterer L, Garhofer G How can blood flow be measured? *Surv Ophthalmol*. 2007; 52(Suppl 2): S134–138. 17998038 10.1016/j.survophthal.2007.08.008

[14] Wei X, Balne PK, Meissner KE, Barathi VA, Schmetterer L, Agrawal R Assessment of flow dynamics in retinal and choroidal microcirculation. *Surv Ophthalmol*. 2018; 63: 646–664. 29577954 10.1016/j.survophthal.2018.03.003

[15] Werkmeister RM, Dragostinoff N, Palkovits S, et al. Measurement of absolute blood flow velocity and blood flow in the human retina by dual-beam bidirectional Doppler Fourier-domain optical coherence tomography. *Invest Ophthalmol Vis Sci*. 2012; 53: 6062–6071. 22893675 10.1167/iovs.12-9514

[16] Nagel E, Vilser W Autoregulative behavior of retinal arteries and veins during changes of perfusion pressure: a clinical study. *Graefes Arch Clin Exp Ophthalmol*. 2004; 242: 13–17. 14648137 10.1007/s00417-003-0663-3

[17] Riva CE, Grunwald JE, Petrig BL Autoregulation of human retinal blood flow. An investigation with laser Doppler velocimetry. *Invest Ophthalmol Vis Sci*. 1986; 27: 1706–1712. 2947873

[18] Schulte K, Wolf S, Arend O, Harris A, Henle C, Reim M Retinal hemodynamics during increased intraocular pressure. *Ger J Ophthalmol*. 1996; 5: 1–5. 8646172

[19] Leske MC Ocular perfusion pressure and glaucoma: clinical trial and epidemiologic findings. *Curr Opin Ophthalmol*. 2009; 20: 73–78. 19240538 10.1097/ICU.0b013e32831eef82 PMC2662722

[20] Ulrich WD, Ulrich C Oculo-oscillo-dynamography: a diagnostic procedure for recording ocular pulses and measuring retinal and ciliary arterial blood pressures. *Ophthalmic Res*. 1985; 17: 308–317. 4069567 10.1159/000265391

[21] Doblhoff-Dier V, Schmetterer L, Vilser W, et al. Measurement of the total retinal blood flow using dual beam Fourier-domain Doppler optical coherence tomography with orthogonal detection planes. *Biomed Opt Express*. 2014; 5: 630–642. 24575355 10.1364/BOE.5.000630 PMC3920891

[22] Leitgeb RA, Werkmeister RM, Blatter C, Schmetterer L Doppler optical coherence tomography. *Prog Retin Eye Res*. 2014; 41: 26–43. 24704352 10.1016/j.preteyeres.2014.03.004 PMC4073226

[23] Fondi K, Aschinger GC, Bata AM, et al. Measurement of retinal vascular caliber from optical coherence tomography phase images. *Invest Ophthalmol Vis Sci*. 2016; 57: Oct121–129. 27409462 10.1167/iovs.15-18476

[24] Luksch A, Polska E, Imhof A, et al. Role of NO in choroidal blood flow regulation during isometric exercise in healthy humans. *Invest Ophthalmol Vis Sci*. 2003; 44: 734–739. 12556406 10.1167/iovs.02-0177

[25] Fuchsjager-Mayrl G, Luksch A, Malec M, Polska E, Wolzt M, Schmetterer L Role of endothelin-1 in choroidal blood flow regulation during isometric exercise in healthy humans. *Invest Ophthalmol Vis Sci*. 2003; 44: 728–733. 12556405 10.1167/iovs.02-0372

[26] Riva CE, Sinclair SH, Grunwald JE Autoregulation of retinal circulation in response to decrease of perfusion pressure. *Invest Ophthalmol Vis Sci*. 1981; 21: 34–38. 7251300

[27] Riva CE, Titze P, Hero M, Movaffaghy A, Petrig BL Choroidal blood flow during isometric exercises. *Invest Ophthalmol Vis Sci*. 1997; 38: 2338–2343. 9344357

[28] Schmidl D, Boltz A, Kaya S, et al. Role of nitric oxide in optic nerve head blood flow regulation during isometric exercise in healthy humans. *Invest Ophthalmol Vis Sci*. 2013; 54: 1964–1970. 23439596 10.1167/iovs.12-11406

[29] Chiquet C, Lacharme T, Riva C, et al. Continuous response of optic nerve head blood flow to increase of arterial blood pressure in humans. *Invest Ophthalmol Vis Sci*. 2014; 55: 485–491. 24355824 10.1167/iovs.13-12975

[30] Movaffaghy A, Chamot SR, Petrig BL, Riva CE Blood flow in the human optic nerve head during isometric exercise. *Exp Eye Res*. 1998; 67: 561–568. 9878218 10.1006/exer.1998.0556

[31] Garhofer G, Resch H, Weigert G, Lung S, Simader C, Schmetterer L Short-term increase of intraocular pressure does not alter the response of retinal and optic nerve head blood flow to flicker stimulation. *Invest Ophthalmol Vis Sci*. 2005; 46: 1721–1725. 15851574 10.1167/iovs.04-1347

[32] Garhofer G, Bek T, Boehm AG, et al. Use of the retinal vessel analyzer in ocular blood flow research. *Acta Ophthalmol*. 2010; 88: 717–722. 19681764 10.1111/j.1755-3768.2009.01587.x

[33] Sakai J, Minamide KJ, Nakamura S, et al. Retinal arteriole pulse waveform analysis using a fully-automated Doppler optical coherence tomography flowmeter: a pilot study. *Transl Vis Sci Technol*. 2019; 8: 13. 10.1167/tvst.8.3.13 PMC6504205 31110914

[34] Costa VP, Harris A, Anderson D, et al. Ocular perfusion pressure in glaucoma. *Acta Ophthalmol*. 2014; 92: e252–266. 24238296 10.1111/aos.12298

[35] Fondi K, Wozniak PA, Howorka K, et al. Retinal oxygen extraction in individuals with type 1 diabetes with no or mild diabetic retinopathy. *Diabetologia*. 2017; 60: 1534–1540. 28547132 10.1007/s00125-017-4309-0 PMC5491565

